# TOR-Dependent and -Independent Pathways Regulate Autophagy in *Arabidopsis thaliana*

**DOI:** 10.3389/fpls.2017.01204

**Published:** 2017-07-11

**Authors:** Yunting Pu, Xinjuan Luo, Diane C. Bassham

**Affiliations:** ^1^Department of Genetics, Development and Cell Biology, Iowa State University, Ames IA, United States; ^2^Interdepartmental Genetics Program, Iowa State University, Ames IA, United States; ^3^College of Life Sciences, Northwest A&F University Yangling, China; ^4^Plant Sciences Institute, Iowa State University, Ames IA, United States

**Keywords:** autophagy, TOR signaling, stress responses, auxin, Arabidopsis

## Abstract

Autophagy is a critical process for recycling of cytoplasmic materials during environmental stress, senescence and cellular remodeling. It is upregulated under a wide range of abiotic stress conditions and is important for stress tolerance. Autophagy is repressed by the protein kinase target of rapamycin (TOR), which is activated in response to nutrients and in turn upregulates cell growth and translation and inhibits autophagy. Down-regulation of TOR in *Arabidopsis thaliana* leads to constitutive autophagy and to decreased growth, but the relationship to stress conditions is unclear. Here, we assess the extent to which TOR controls autophagy activation by abiotic stress. Overexpression of *TOR* inhibited autophagy activation by nutrient starvation, salt and osmotic stress, indicating that activation of autophagy under these conditions requires down-regulation of TOR activity. In contrast, TOR overexpression had no effect on autophagy induced by oxidative stress or ER stress, suggesting that activation of autophagy by these conditions is independent of TOR function. The plant hormone auxin has been shown previously to up-regulate TOR activity. To confirm the existence of two pathways for activation of autophagy, dependent on the stress conditions, auxin was added exogenously to activate TOR, and the effect on autophagy under different conditions was assessed. Consistent with the effect of TOR overexpression, the addition of the auxin NAA inhibited autophagy during nutrient deficiency, salt and osmotic stress, but not during oxidative or ER stress. NAA treatment was unable to block autophagy induced by a TOR inhibitor or by a mutation in the TOR complex component *RAPTOR1B*, indicating that auxin is upstream of TOR in the regulation of autophagy. We conclude that repression of auxin-regulated TOR activity is required for autophagy activation in response to a subset of abiotic stress conditions.

## Introduction

Plants have evolved many response mechanisms to adapt to various growth conditions, including abiotic stresses. One such mechanism is autophagy, a major pathway for degradation and recycling of cytoplasmic materials in all eukaryotes (Liu and Bassham, 2012; Yang and Bassham, 2015). Autophagy is active at a low basal level even under normal conditions, and numerous human diseases are linked to autophagy defects, including cancer and various neurodegenerative diseases such as Parkinson’s, Huntington’s, and Alzheimer’s diseases (Cai et al., 2016; Davidson and Vander Heiden, 2017). In plants, autophagy functions in the response to both abiotic and biotic stress, and is induced during senescence and nutrient deficiency (Doelling et al., 2002; Hanaoka et al., 2002), salt and drought stresses (Liu et al., 2009), oxidative stress (Xiong et al., 2007b), endoplasmic reticulum (ER) stress (Liu et al., 2012), and pathogen infection (Liu et al., 2005).

When autophagy is activated, a double-membrane cup-shaped structure named a phagophore is formed. The phagophore expands to form a double-membrane vesicle called an autophagosome, while engulfing cellular components to be degraded. Autophagosomes are delivered to and fuse with lysosomes in mammalian cells or the vacuole in plant or yeast cells, where the cargo is degraded into small molecules by vacuolar hydrolases and recycled (Yang and Bassham, 2015). Studies in yeast have identified more than 30 autophagy-related (*ATG*) genes, many of which have also been found in plants (Tsukada and Ohsumi, 1993; Yang and Bassham, 2015). A key protein involved in autophagosome formation is ATG8, which can be used as a marker for autophagosomes when fused with a fluorescent protein (Yoshimoto et al., 2004; Contento et al., 2005). ATG8 is attached to the autophagosome membrane through a covalent bond to phosphatidylethanolamine (PE) via two ubiquitin-like conjugation systems that include the E1-like activating enzyme ATG7 (Ichimura et al., 2000). Knockout of *ATG7* therefore prevents autophagosome formation, leading to plants being hypersensitive to both abiotic and biotic stress conditions (Doelling et al., 2002; Lenz et al., 2011; Zhou et al., 2013).

The target of rapamycin (TOR) complex is a key regulator of autophagy, and is composed of TOR itself and two binding partners, regulatory-associated protein of TOR (RAPTOR), and Lethal with Sec Thirteen 8 (LST8) (Yang et al., 2013; Dobrenel et al., 2016). TOR is a Ser/Thr protein kinase in the phosphatidylinositol-3-kinase (PI3K) – related kinase (PIKK) family (Noda and Ohsumi, 1998; Menand et al., 2002), whereas RAPTOR recruits substrates to the complex for phosphorylation by TOR (Hara et al., 2002), and LST8 stabilizes the complex (Yang et al., 2013). The TOR signaling pathway both positively regulates cell growth and metabolism and negatively regulates autophagy in yeast, mammals, and plants (Dobrenel et al., 2016). In *Arabidopsis thaliana*, a null mutation in *TOR* is embryo lethal (Menand et al., 2002), whereas decreased *TOR* expression due to RNA interference leads to autophagy induction (Liu and Bassham, 2010), and arrested plant growth and development (Deprost et al., 2007). Active-site TOR inhibitors (asTORis) that disrupt TOR activity by competition for ATP-binding also result in plant growth defects (Montane and Menand, 2013). Consistent with this, overexpression of *TOR* enhances growth and osmotic stress resistance (Deprost et al., 2007; Ren et al., 2011).

Two *RAPTOR* genes exist in Arabidopsis, *RAPTOR1A* and *RAPTOR1B* (Anderson et al., 2005; Deprost et al., 2005). A *raptor1b* null mutant has growth defects, including delayed leaf initiation and growth, late bolting and flowering, and short roots, while *raptor1a* knock out mutants have no major developmental phenotypes, possibly due to the higher expression of *RAPTOR1B* in most plant tissues (Anderson et al., 2005; Deprost et al., 2005). A *raptor1a raptor1b* double knockout mutant has minimal meristem growth, indicating that RAPTOR1A and RAPTOR1B might have some distinct functions, but is not embryo-lethal, and TOR must therefore retain some of its function in the absence of RAPTOR (Anderson et al., 2005). Two *LST8* genes have also been identified in Arabidopsis, *LST8-1* and *LST8-2*, although only *LST8-1* appears to be expressed (Moreau et al., 2012). The null mutant *lst8-1* has strong growth defects and impaired adaptation to long day conditions (Moreau et al., 2012). Mutation of *lst8-1* or *raptor1b*, or disruption of TOR activity with asTORis, causes hypersensitivity to abscisic acid (ABA) and decreased ABA synthesis (Kravchenko et al., 2015), indicating that the TOR complex may also play a role in hormone signaling.

Target of rapamycin signals through phosphorylation of downstream substrates (Raught et al., 2001; Ren et al., 2011). Several TOR substrates have been identified in Arabidopsis, including the p70 ribosomal protein S6 kinase (S6K) (Mahfouz et al., 2006; Xiong and Sheen, 2012), the E2Fa transcription factor, which activates cell cycle genes (Xiong et al., 2013; Li et al., 2017), and TAP46, a regulatory subunit of protein phosphatase type 2A (PP2A), which was suggested to regulate plant growth and autophagy (Yorimitsu et al., 2009; Ahn et al., 2011). Arabidopsis has two S6K paralogs with 87% sequence identity, S6K1 and S6K2, both of which are phosphorylated by TOR. The activity of plant S6Ks increases in response to auxin and cytokinins (Turck et al., 2004).

Upstream regulation of TOR signaling in plants is still poorly understood. Auxin can enhance TOR activity to promote the translation reinitiation of mRNAs via S6K1, and deficiency in TOR signaling impaired auxin-mediated root gravitropism (Schepetilnikov et al., 2013). Auxin regulation is mediated by the small GTPase ROP2, which directly binds to and activates TOR (Li et al., 2017; Schepetilnikov et al., 2017). These studies indicate that auxin might regulate plant growth, development and stress responses through the TOR signaling pathway. In this study, we first confirm that the TOR complex is a negative regulator of autophagy in Arabidopsis, and demonstrate a role for RAPTOR1B in this regulation. We show that TOR regulates autophagy induced by nutrient starvation, salt or osmotic stress, but not oxidative or ER stress, indicating that TOR-dependent and -independent pathways for regulation of autophagy exist in plants. In addition, exogenous auxin has similar effects on stress-induced autophagy as TOR overexpression, suggesting a mechanism by which auxin interfaces with stress responses in plants through regulation of TOR activity.

## Materials and Methods

### Plant Materials and Growth Conditions

*Arabidopsis thaliana* seeds of WT (Col-0) or other indicated genotypes were sterilized with 33% (v/v) bleach and 0.1% (v/v) Triton X-100 (Sigma) for 20 min, followed by five washes of 5 min each with sterile water. Sterilized seeds were stored at 4°C in darkness for at least 2 days to allow stratification before plating on solid ½ MS medium (2.22 g/L Murashige-Skoog vitamin and salt mixture [Caisson Laboratory, MSP09], 1% [w/v] sucrose, 0.6% [w/v] Phytoblend agar [Caisson Laboratory], 2.4 mM 2-morphinolino-ethanesulfonic acid [MES, Sigma], pH 5.7). Seedlings were grown under long-day conditions (16 h light) at 22°C for 7 days. Plants for transient expression in leaf protoplasts were grown in soil in a humidity-controlled growth chamber with 50% humidity at 20–23°C under long-day conditions for 4–6 weeks. T-DNA insertion mutants used in this study are: *raptor1a* (SALK_043920c), *raptor1b* (SALK_078159) (Anderson et al., 2005), S7817 (SALK_147817), G166 (GABI_166C06), G548 (GABI_548G07) (Deprost et al., 2007), and *atg7* (GABI_655B06) (Chung et al., 2010). Transgenic plants used in this study are: *GFP-ATG8e* (Xiong et al., 2007b), *TOR-OE1* and *TOR-OE2* (Ren et al., 2011).

### Stress and Drug Treatments

For sucrose and nitrogen starvation, 7-day-old seedlings grown on solid ½ MS medium were transferred to solid ½ MS medium lacking sucrose or nitrogen for an additional 3 days (Doelling et al., 2002). Sucrose starvation plates were kept in the dark after transfer. For salt and mannitol treatment, 7-day-old seedlings grown on solid ½ MS medium were transferred to liquid ½ MS medium with 0.16 M NaCl or 0.35 M mannitol for 6–8 h. For oxidative and ER stress, 7-day-old seedlings grown on solid ½ MS medium were transferred to liquid ½ MS medium with 5 mM H_2_O_2_ (Sigma) for 2–3 h, or with 2 mM dithiothreitol (DTT, Fisher) or 5 μg/mL tunicamycin (Sigma) for 6–8 h. For AZD8055 treatment, 7-day-old seedlings grown on solid ½ MS medium were transferred to solid ½ MS medium with 2 μM AZD8055 (LC Laboratories) for 1 day, or liquid ½ MS medium with 1 μM AZD8055 for 2–3 h.

For auxin treatment, 7-day-old seedlings were transferred to solid ½ MS medium supplemented with 20 nM 1-naphthaleneacetic acid (NAA, Sigma-Aldrich, N0640) with or without starvation for an additional 3 days, or in liquid ½ MS medium with 20 nM NAA for 6–8 h with or without stress treatments as described above. For BTH treatment, 7-day-old seedlings were transferred to liquid ½ MS medium supplemented with 100 μM acibenzolar-*S*-methyl (BTH, Sigma-Aldrich, 32820) with or without 20 nM NAA for 8 h.

For concanamycin A treatment, 7-day-old GFP-ATG8e seedlings were transferred to liquid ½ MS medium with DMSO or 1 μM concanamycin A (Sigma) with or without other stress or drug treatments for 6–8 h.

### Autophagy Detection by Fluorescence Microscopy

Arabidopsis seedling roots were stained with monodansylcadaverine (MDC) as described previously (Contento et al., 2005). MDC-stained seedlings were observed with a Zeiss Axio Imager.A2 upright microscope (Zeiss) equipped with Zeiss Axiocam BW/color digital cameras using a DAPI-specific filter at the Iowa State University Microscopy and Nanoimaging Facility. GFP-ATG8e transgenic seedlings were observed and photographed with the same microscope system with a GFP-specific filter. Cells within the root elongation zone were photographed and the number of autophagosomes in each image was counted and averaged from at least 10 images per sample. Confocal microscopy images of autophagosomes in root cells and leaf protoplasts were taken using a Leica SP5 × MP confocal/multiphoton microscope system (Leica) with a 63x/1.4 oil immersion objective at the Iowa State University Roy J. Carver High Resolution Microscopy Facility (Pu and Bassham, 2016).

### Transient Expression in Protoplasts

GFP-ATG8e was transiently expressed in Arabidopsis leaf protoplasts as previously described (Sheen, 2002; Liu et al., 2012). 25–30 μg of GFP-ATG8e plasmid DNA was introduced into protoplasts using 40% (w/v) polyethylene glycol (PEG, Sigma-Aldrich). Protoplasts were washed and incubated in W5 solution (154 mM NaCl, 125 mM CaCl_2_, 5 mM KCl, 2 mM MES, pH 5.7). For starvation treatment, protoplasts were incubated in W5 solution without sucrose or with 0.5% (w/v) sucrose as control at room temperature in darkness for 2 days in total. For other stress treatments, protoplasts were incubated in W5 solution with treatments as described in the Stress and Auxin Treatment section. Protoplasts were observed by fluorescence microscopy (Nikon Eclipse E200) using a FITC filter, and protoplasts with more than three visible autophagosomes were counted as active for autophagy (Yang et al., 2016). A total of 100 protoplasts were observed per genotype for each condition, and the percentage of protoplasts with induced autophagy was calculated and averaged from three independent experimental replicates.

### Generation of RAPTOR1B Construct

The RAPTOR1B cDNA sequence was divided into two fragments, and each fragment was amplified from Col-0 cDNA using CloneAmp HiFi PCR Premix (Takara). The 5′ fragment of RAPTOR1B was amplified with forward primer 5′-CACCGAGCTCGAATTCATGGCATTAGGAGACTTAATGGTGTCTC-3′ (inserted SacI restriction site underlined), and reverse primer 5′-GTCAAACCCAATATCAAGCAAGGTACCCA-3′, digested with SacI and KpnI (within the RAPTOR1B cDNA sequence), and ligated into the pPZP212 binary vector (Hajdukiewicz et al., 1994; Li et al., 2009), which has a 35S promoter sequence at the 5′ end and a MYC tag sequence at the 3′ end of the insert. The 3′ fragment was amplified with forward primer 5′-TGGGTACCTTGCTTGATATTGGGTTTGAC-3′ and reverse primer 5′-CACCGTCGACTCTTGCTTGCGAGTTGTCGTGGGTG-3′ (inserted SalI restriction site underlined), digested with KpnI and SalI, and ligated into the pPZP212 vector containing the 5′ fragment to complete the full sequence. The entire construct was confirmed by sequencing.

### Accession Numbers

Sequence data from this article can be found in the Arabidopsis Genome Initiative under the following accession numbers: TOR, AT1G50030; RAPTOR1A, AT5G01770, RAPTOR1B, AT3G08850; ATG8e, AT2G45170; ATG7, AT5G45900.

## Results

### Inhibition of TOR Signaling Leads to Constitutive Autophagy

**FIGURE 1:**
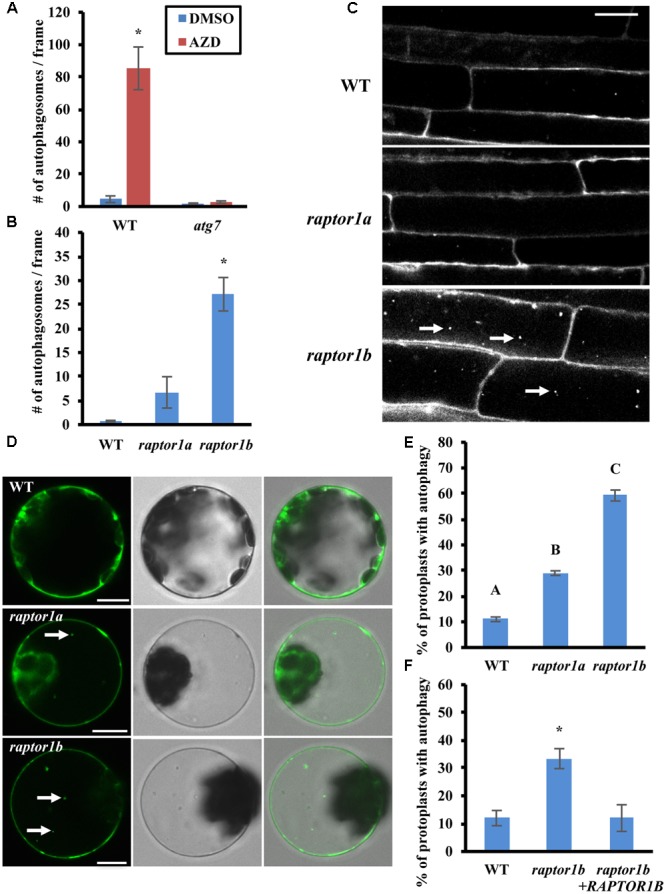
Inhibition of TOR or RAPTOR leads to constitutive autophagy. **(A)** The TOR inhibitor AZD8055 induces autophagy. 7-day-old WT (Col-0) and *atg7* mutant seedlings were treated with DMSO or AZD8055 (AZD) for 2–3 h, stained with MDC and then observed and imaged by fluorescence microscopy. The number of puncta in each image was counted and averaged from at least 10 images per genotype for each condition. **(B,C)** Autophagy is induced in *raptor1b* mutant root cells under standard growth conditions. **(B)** 7-day-old WT, *raptor1a* and *raptor1b* knockout mutant seedlings were stained with MDC and observed by fluorescence microscopy. The number of puncta in each image was quantified as in **(A)**. **(C)** Representative confocal images of MDC-stained WT, *raptor1a* and *raptor1b* mutant seedlings. MDC-stained autophagosomes appear as white puncta within cells as indicated by white arrows. Scale bar = 20 μm. **(D,E)** Leaf protoplasts of *raptor1a* and *raptor1b* mutants have constitutive autophagy. **(D)** Transient expression of a GFP-ATG8e fusion protein in leaf protoplasts of WT and *RAPTOR* mutants, observed by confocal microscopy. GFP-tagged autophagosomes appear as green puncta within leaf protoplasts in the left column as indicated by white arrows. The middle and right columns show DIC and merged images respectively. Scale bar = 10 μm. **(E)** Quantification of D. Protoplasts were observed using epifluorescence microscopy. The percentage of protoplasts with more than three visible GFP-tagged autophagosomes was calculated, with 100 protoplasts observed per genotype for each condition. **(F)** Expression of the *RAPTOR1B* cDNA complements the *raptor1b* constitutive autophagy phenotype. A GFP-ATG8e fusion protein was transiently expressed in *raptor1b* mutant leaf protoplasts with or without full-length *RAPTOR1B*, expressed from a 35S constitutive promoter, or in WT protoplasts as a control. Protoplasts were observed using epifluorescence microscopy. The percentage of protoplasts with more than three visible GFP-tagged autophagosomes was quantified as in **(E)**. For all graphs, error bars indicate means ± standard error (SE) from three independent replicates. Asterisks or different letters indicate statistically significant differences (*P* < 0.05) using Student’s *t*-test compared with WT under control conditions.

We have shown previously that decreased *TOR* expression via RNA interference induces autophagy in Arabidopsis, suggesting that TOR is a negative regulator of autophagy in plants (Liu and Bassham, 2010). To confirm that autophagy is induced by inhibition of TOR kinase activity (Montane and Menand, 2013), we examined autophagy activity after application of the asTORis AZD8055 (Dong et al., 2015). WT and *atg7* seedlings, a previously characterized knockout mutant that is unable to form autophagosomes (Doelling et al., 2002), were grown under standard conditions for 7 days, followed by 1 μM AZD8055 treatment in liquid ½ MS medium for 2–3 h. Roots of seedlings were stained with monodansylcadaverine (MDC), an acidotropic dye that can stain autophagosomes (Biederbick et al., 1995; Contento et al., 2005), and examined by fluorescence microscopy. Autophagosomes appear as rapidly moving fluorescent puncta, and the number of visible puncta in each image were counted for quantification. As expected, compared to the basal level of autophagy in the control, inhibition of TOR activity by AZD8055 led to a significant increase in the number of autophagosomes (**Figure 1A**), while no autophagosomes were detected in the *atg7* mutant. This confirmed that TOR negatively regulates autophagy in Arabidopsis, and that the kinase activity of TOR is critical for this regulation.

Previous studies have shown that down-regulation of *TOR* or its binding partners *RAPTOR* and *LST8* leads to defects in plant growth and development (Anderson et al., 2005; Deprost et al., 2007; Moreau et al., 2012; Montane and Menand, 2013), suggesting that RAPTOR and LST8 are critical for TOR-regulated plant growth. To test whether inhibition of TOR complex activity by disruption of *RAPTOR* also induces autophagy, WT, *raptor1a* and *raptor1b* knockout mutant seedlings (Anderson et al., 2005) were grown on ½ MS medium with sucrose for a week, and autophagy in root cells was examined by MDC staining followed by fluorescence microscopy (**Figures 1B,C**). Compared to the basal level of autophagy in WT seedlings, the number of autophagosomes in the *raptor1a* mutant appeared slightly higher, but this difference was not statistically significant, possibly due to the variability between seedlings. The *raptor1b* mutant had a significantly higher number of autophagosomes, suggesting that the *raptor1b* mutant has constitutive autophagy, and that *RAPTOR1A* and *RAPTOR1B* may not function equally in autophagy regulation.

To confirm that the *raptor1b* mutant has increased basal autophagy under standard conditions, the autophagosome marker GFP-ATG8e was expressed transiently in WT, *raptor1a* and *raptor1b* leaf protoplasts (**Figure 1D**). The GFP-ATG8e fusion protein has been used extensively as a specific marker of autophagosomes and autophagic bodies (Yoshimoto et al., 2004; Contento et al., 2005; Pu and Bassham, 2016), and active autophagy is defined as more than three visible autophagosomes in a protoplast (Yang et al., 2016). WT protoplasts maintain a basal level of autophagy with a low percentage with active autophagy. Consistent with the MDC staining results, the percentage of *raptor1b* protoplasts with active autophagy was significantly higher than that of WT protoplasts (**Figure 1E**). However, *raptor1a* also had a significantly higher percentage of active autophagy in leaf protoplasts, although significantly lower than *raptor1b*. *RAPTOR1A* may therefore be more important for autophagy regulation in leaves than in roots.

To confirm that the constitutive autophagy in the *raptor1b* mutant is specifically due to the mutation in *RAPTOR1B*, the *RAPTOR1B* cDNA was transiently expressed under a 35S promoter together with GFP-ATG8e in *raptor1b* knock out mutant leaf protoplasts. Autophagy was assessed as described above using fluorescence microscopy (**Figure 1F**). Note that the number of autophagosomes in the *raptor1b* mutant is variable between experiments, depending most likely on the growth conditions and age of the plants, room temperature, etc. The percentage of protoplasts with active autophagy in the *raptor1b* mutant expressing the *RAPTOR1B* cDNA was substantially lower than for the mutant protoplasts alone, and was not significantly different from WT. This indicates that the increased basal autophagy observed in the *raptor1b* mutant was suppressed by expression of the *RAPTOR1B* cDNA, confirming that the constitutive autophagy phenotype is indeed due to the disruption of *RAPTOR1B*.

### Overexpression of TOR Blocks Autophagy upon Starvation, Salt and Drought Stress

**FIGURE 2:**
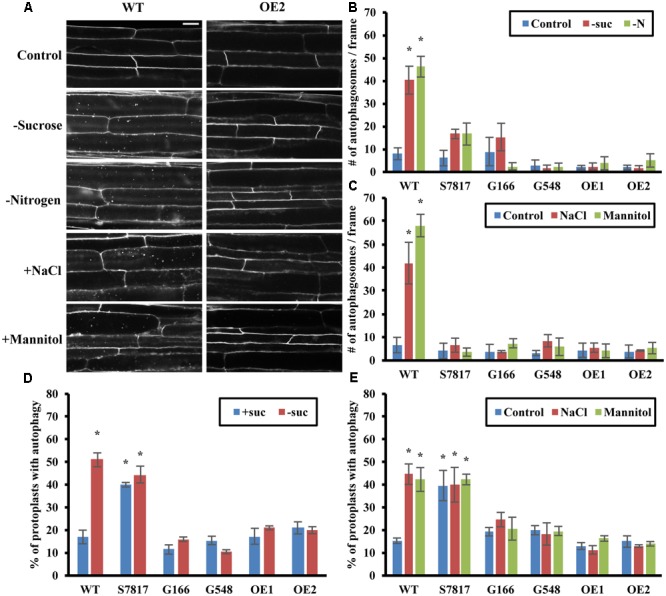
Overexpression of TOR blocks autophagy induced by nutrient starvation, salt and osmotic stresses. **(A)** Representative confocal images of MDC-stained WT and *TOR-OE2* seedlings after the indicated stress treatment. For nutrient starvation, 7-day-old seedlings of WT and *TOR-OE2* transgenic lines were transferred to solid ½ MS medium for an additional 3 days with or without nitrogen in the light, or without sucrose in the dark. For salt and osmotic stress, 7-day-old WT and *TOR-OE2* seedlings were transferred to liquid ½ MS medium plus or minus 0.16 M NaCl or 0.35 M mannitol for 6–8 h. Scale bar = 20 μm. **(B,C)** Quantification of autophagosome number in WT and TOR overexpression lines after sucrose or nitrogen starvation **(B)**, salt, or osmotic stress **(C)**, treated as in **(A)**. MDC-stained autophagosomes were observed by fluorescence microscopy and photographed. The average number of autophagosomes was calculated from 10 images per genotype for each condition. **(D,E)** TOR overexpression lines fail to activate autophagy under sucrose starvation **(D)**, salt or osmotic stress **(E)** in leaf protoplasts. A GFP-ATG8e fusion protein was transiently expressed in leaf protoplasts of WT and TOR overexpression lines. Protoplasts were incubated in the dark plus or minus 0.5% (w/v) sucrose for 2 days **(D)**, or plus or minus 0.16 M NaCl or 0.35 M mannitol for 1 day **(E)**. Protoplasts were observed using fluorescence microscopy. The percentage of protoplasts with more than three visible GFP-tagged autophagosomes was calculated from 100 protoplasts observed per genotype for each condition. For all graphs, error bars indicate means ± SE from three independent replicates. Asterisks indicate statistically significant differences (*P* < 0.05) using Student’s *t*-test compared with WT under control conditions.

Previous studies and our data have shown that the TOR complex negatively regulates autophagy in Arabidopsis (Liu and Bassham, 2010) (**Figure 1**), but the conditions under which TOR is important in plants are unknown. In other organisms TOR is well-described as regulating autophagy in response to nutrients (Dobrenel et al., 2016), with a decrease in TOR activity during nutrient deficiency leading to activation of autophagy. We therefore hypothesized that overexpression of TOR might prevent activation of autophagy by nutrient deficiency, and that autophagy induction by other stresses might be TOR-independent. To test this hypothesis, we obtained several previously characterized Arabidopsis lines with T-DNA insertions in the TOR upstream region (S7817, G166, and G548) and two transgenic lines with TOR expressed from a 35S promotor (*TOR-OE1* and *TOR-OE2*). All lines have overexpression of TOR and enhanced growth (Deprost et al., 2007; Ren et al., 2011), with the exception of S7817, which has decreased TOR expression in leaves and overexpression of TOR in roots (Deprost et al., 2007). Seeds of WT and the five TOR overexpression lines were germinated and grown on solid ½ MS medium plus sucrose for 1 week, followed by transfer to solid ½ MS medium plus or minus nitrogen in the light, or minus sucrose in the dark for an additional 3 days. Autophagosomes were detected by MDC staining followed by fluorescence microscopy (**Figures 2A,B**). Representative images of one of the TOR overexpression lines are shown in **Figure 2A**. Quantification of autophagosomes indicated that both WT and the TOR overexpression lines had a low basal level of autophagy under control conditions. The average number of autophagosomes in WT seedlings after sucrose or nitrogen starvation was significantly higher than in control conditions, whereas the TOR overexpression lines had no significant activation of autophagy. This indicates that overexpression of TOR can repress autophagy induced by nutrient starvation, suggesting that repression of TOR activity is required for activation of autophagy in response to nutrient depletion.

While previous studies have shown that TOR is involved in nutrient sensing (Dobrenel et al., 2016), the extent to which TOR regulates stress responses other than nutrient deficiency is not known, although a link to osmotic stress resistance in Arabidopsis has been suggested (Mahfouz et al., 2006; Deprost et al., 2007). Autophagy is activated in Arabidopsis by salt and osmotic stresses (Liu et al., 2009). Therefore, we also tested whether overexpression of TOR affects autophagy induced by salt or osmotic stress (**Figures 2A,C**). WT and the TOR overexpression lines were germinated and grown on solid ½ MS medium for 1 week, and then transferred to liquid ½ MS medium containing 0.16 M NaCl or 0.35 M mannitol for 6–8 h. Autophagy in seedling roots was assayed by MDC staining followed by fluorescence microscopy (**Figure 2C**). Autophagy in salt or mannitol treated WT seedlings was significantly higher than the basal level of autophagy seen under control conditions. As for nutrient deficiency, autophagy was not induced in TOR overexpression lines under salt or osmotic stress, indicating that TOR can also repress autophagy induced by these stresses.

To confirm these results, we measured autophagy by transient expression of GFP-ATG8e in leaf protoplasts from WT and TOR overexpressing plants under sucrose starvation, salt and osmotic stresses (**Figures 2D,E**). As the protoplast incubation buffer contains nitrogen, it was not possible to test nitrogen deficiency using our standard protocol. After transformation with GFP-ATG8e constructs, protoplasts were incubated with or without sucrose for 2 days (**Figure 2D**), or plus or minus 0.16 M NaCl or 0.35 M mannitol for 1 day (**Figure 2E**), after which autophagy was observed using fluorescence microscopy. The percentage of protoplasts with active autophagy was calculated, with 100 protoplasts observed per genotype for each condition. WT and TOR overexpression lines had a low level of autophagy under control conditions, except for the S7817 line which had constitutive activation of autophagy. In this line, TOR expression is decreased in leaves, potentially explaining this observation (Deprost et al., 2007). While WT protoplasts had a significantly higher level of autophagy under sucrose starvation, salt and osmotic stresses, autophagy in the TOR overexpression lines, with the exception of S7817, remained at a low basal level indistinguishable from that in control conditions. We conclude that TOR is a regulator of autophagy in response to salt and osmotic stress in addition to nutrient deficiency.

### Overexpression of TOR has No Effect on Oxidative Stress- or ER Stress-Induced Autophagy

**FIGURE 3:**
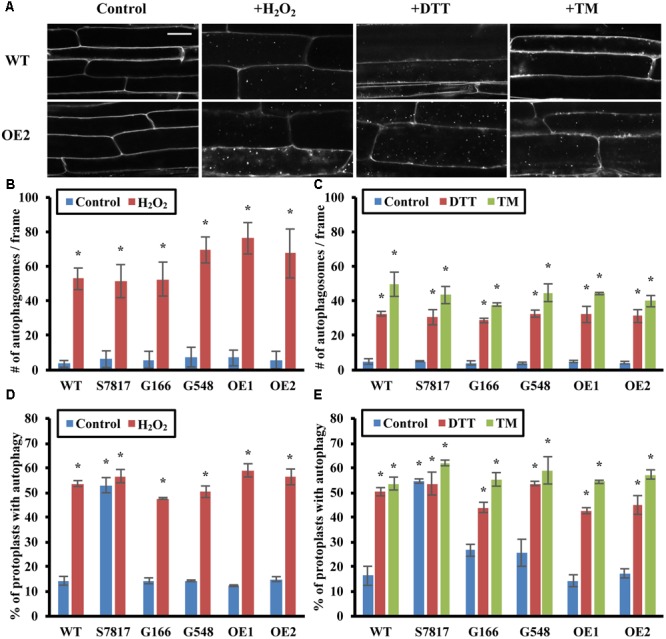
Overexpression of TOR has no effect on oxidative stress- or ER stress- induced autophagy. **(A)** Confocal microscopy of MDC-stained WT and *TOR-OE2* seedlings after the indicated stress treatment. 7-day-old WT and TOR overexpression transgenic seedlings were transferred to liquid ½ MS medium with or without 5 mM H_2_O_2_ for 2–3 h for oxidative stress, or 2 mM DTT or 5 μg/mL tunicamycin (TM) for 6–8 h for ER stress. Scale bar = 20 μm. **(B,C)** Quantification of autophagosome number in WT and TOR overexpression lines under oxidative stress **(B)** or ER stress **(C)** treated as in A. MDC-stained autophagosomes were observed by fluorescence microscopy and photographed. The average number of autophagosomes was calculated from 10 images per genotype for each condition. **(D,E)** Autophagy was induced in leaf protoplasts of TOR overexpression lines under oxidative stress **(D)** or ER stress **(E)**. A GFP-ATG8e fusion protein was transiently expressed in leaf protoplasts of WT and TOR overexpression lines. Protoplasts were incubated in the dark plus or minus 5 mM H_2_O_2_ for 2–3 h **(D)**, or 2 mM DTT or 5 μg/mL tunicamycin (TM) for 6–8 h **(E)**. Protoplasts were observed using fluorescence microscopy. The percentage of protoplasts with more than three visible GFP-tagged autophagosomes was calculated from 100 protoplasts observed per genotype for each condition. For all graphs, error bars indicate means ± SE from three independent replicates. Asterisks indicate statistically significant differences (*P* < 0.05) using Student’s *t*-test compared with WT under control conditions.

Autophagy is also induced by oxidative stress and ER stress in plants (Xiong et al., 2007b; Liu et al., 2012; Yang et al., 2016). Oxidative stress is triggered when cells accumulate excessive reactive oxygen species (ROS), and oxidized proteins and lipids are degraded through autophagy (Xiong et al., 2007a,b). ER stress is generated when unfolded or misfolded proteins exceed the capacity of protein folding or degradation systems, causing accumulation of proteins in the ER (Howell, 2013). It can be triggered by heat stress, or experimentally by chemicals such as dithiothreitol (DTT) or tunicamycin (Howell, 2013). To determine whether TOR regulates autophagy upon oxidative or ER stress, 7-day-old WT and TOR overexpression lines were transferred to liquid ½ MS medium plus or minus 5 mM H_2_O_2_ for 2–3 h to cause oxidative stress, or plus 2 mM DTT or 5 μg/mL tunicamycin for 6–8 h to cause ER stress. Autophagy in seedling roots was detected by MDC staining followed by fluorescence microscopy (**Figures 3A–C**). Representative images of one of the TOR overexpression lines are shown in **Figure 3A**. WT and TOR overexpression lines had a low level of autophagy under control conditions, and WT seedlings had significantly higher autophagy induction after oxidative or ER stress treatment. Unlike nutrient, salt or osmotic stresses, TOR overexpression had no effect on autophagy induction, as overexpression lines remained able to activate autophagy under these stresses, suggesting that autophagy is activated via a pathway that does not require inhibition of TOR activity.

To confirm that autophagy remains induced in TOR overexpression lines under oxidative and ER stress, GFP-ATG8e was transiently expressed in leaf protoplasts of WT and TOR overexpression lines. Protoplasts were incubated with or without 5 mM H_2_O_2_, 2 mM DTT, or 5 μg/mL tunicamycin for 1 day, and observed using fluorescence microscopy (**Figures 3D,E**). WT protoplasts had a significantly higher level of autophagy after oxidative or ER stress treatment. In accordance with the MDC staining results, TOR overexpression lines also had a significantly higher percentage of protoplasts with active autophagy after oxidative or ER stress treatment, with no significant difference compared to WT under the same stress conditions. This demonstrates that overexpression of TOR is unable to repress autophagy induced by oxidative or ER stress, suggesting that oxidative- or ER stress-induced autophagy might be regulated through a TOR-independent pathway.

The effects of oxidative stress on autophagy can be difficult to interpret, as autophagy is also triggered by signaling ROS produced by NADPH oxidase (Liu et al., 2009). Salicylic acid (SA) has also been shown to enhance ROS signaling and induce autophagy in plants (Yoshimoto et al., 2009); we therefore tested whether SA-induced autophagy is dependent on TOR. Seven-day-old WT and TOR overexpression lines were transferred to liquid ½ MS medium with 100 μM benzo-(1,2,3)-thiadiazole-7-carbothioic acid *S*-methyl ester (BTH), an SA agonist, or 80% ethanol as control for 8 h. Autophagy in seedling roots was detected by MDC staining followed by fluorescence microscopy (Supplementary Figure 1A). WT and TOR overexpression lines had a low level of autophagy under control conditions, while both WT and TOR overexpression lines had significantly increased autophagy induction upon BTH treatment, suggesting that SA-induced autophagy, as for H_2_O_2_-induced autophagy, is not TOR dependent.

### Auxin Represses Stress-Induced Autophagy through TOR

**FIGURE 4:**
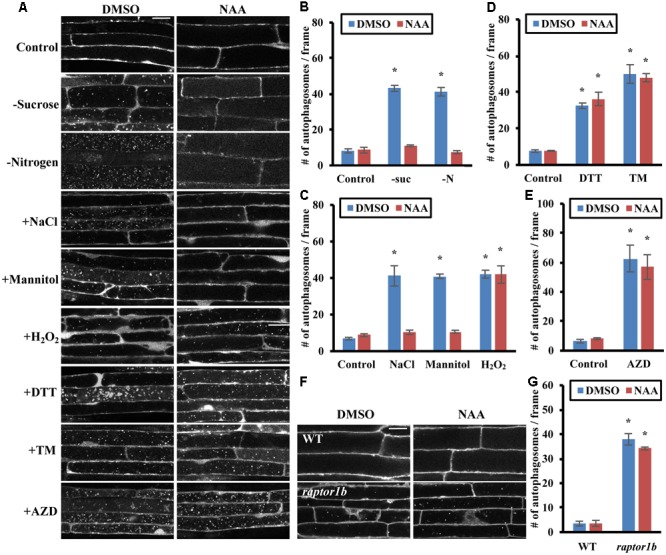
Auxin represses autophagy induced by nutrient starvation, salt and osmotic stress through the TOR signaling pathway. **(A–E)** NAA represses autophagy induced by nutrient starvation, salt and osmotic stresses. **(A)** Representative confocal images of *GFP-ATG8e* transgenic seedlings after NAA and stress treatments. Concanamycin A was included under all conditions to allow accumulation of autophagic bodies inside the vacuole, facilitating visualization. For nutrient starvation, 7-day-old *GFP-ATG8e* seedlings were transferred to solid ½ MS medium plus DMSO or 20 nM NAA for an additional 3 days with or without nitrogen in the light, or without sucrose in the dark. Treated seedlings were then transferred to liquid medium under the same conditions plus 1 μM concanamycin A for an additional 6–8 h. For all other stresses, 7-day-old *GFP-ATG8e* seedlings were transferred to liquid ½ MS medium with 1 μM concanamycin A and DMSO or 20 nM NAA for 6–8 h, together with 0.16 M NaCl, 0.35 M mannitol, 2 mM DTT, or 5 μg/mL tunicamycin (TM) for 6–8 h, or 5 mM H_2_O_2_ or 1 μM AZD8055 during the last 2–3 h of DMSO or NAA treatment. Scale bar = 20 μm. **(B–E)** Quantification of autophagic body number in *GFP-ATG8e* transgenic seedlings under sucrose or nitrogen starvation **(B)**, salt, osmotic stress or oxidative stress **(C)**, ER stress **(D)**, or AZD8055 treatment **(E)**, treated as in **(A)**. GFP-tagged autophagosomes in each condition were observed by fluorescence microscopy and photographed. The number of autophagosomes was counted and averaged from 10 images per genotype for each condition. **(F,G)** Auxin cannot repress the constitutive autophagy seen in a *raptor1b* mutant. **(F)** Representative confocal images of MDC-stained WT and *raptor1b* mutant seedling roots. 7-day-old WT and *raptor1b* seedlings were treated in liquid ½ MS medium with DMSO or 20 nM NAA for 6–8 h. Scale bar = 20 μm. **(G)** Quantification of **(F)**. The average number of autophagosomes was calculated from 10 images per genotype for each condition. For all graphs, error bars indicate means ± SE from three independent replicates. Asterisks indicate statistically significant differences (*P* < 0.05) using Student’s *t*-test compared with WT under control conditions.

Target of rapamycin activity in Arabidopsis can be enhanced by exogenous addition of the auxin 1-naphthaleneacetic acid (NAA) (Schepetilnikov et al., 2013), indicating that auxin might regulate plant growth and development via the TOR signaling pathway. We hypothesized that auxin might repress stress-induced autophagy in plants through the TOR pathway. To confirm the existence of TOR-dependent and -independent pathways for activation of autophagy, NAA was added exogenously to *GFP-ATG8e* transgenic plants to activate TOR, and the effect on autophagy under different conditions was assessed (**Figures 4A–D** and Supplementary Figure 1B). For nutrient deficiency, 7-day-old *GFP-ATG8e* transgenic seedlings were transferred to solid ½ MS medium with or without sucrose or nitrogen and plus 20 nM NAA or DMSO for an additional 3 days. For salt, osmotic and ER stress, and BTH treatment, 7-day-old seedlings were transferred to liquid ½ MS medium plus 0.16 M NaCl, 0.35 M mannitol, 2 mM DTT or 5 μg/mL tunicamycin, or 100 μM BTH and plus 20 nM NAA or DMSO for 6–8 h. For oxidative stress, 7-day-old seedlings were transferred to liquid ½ MS medium plus 20 nM NAA or DMSO for 6–8 h, with 5 mM H_2_O_2_ added only during the last 2–3 h to avoid cell death. To more clearly observe GFP-ATG8e-labeled autophagic bodies in the vacuoles by confocal microscopy, 1 μM concanamycin A was added to block degradation of autophagic bodies prior to imaging of the vacuoles (Dröse et al., 1993; Liu and Bassham, 2010) (**Figure 4A**). In control conditions, root cells had few autophagic bodies within the vacuole, whereas all stresses tested led to accumulation of large numbers of autophagic bodies. In the presence of auxin, autophagic body accumulation was inhibited in nutrient deficiency, salt and osmotic stress, but accumulation was still observed in oxidative and ER stress and upon BTH treatment. These results also indicate that NAA reduces the number of autophagosomes observed by blocking autophagosome formation, rather than by accelerating autophagosome degradation. Autophagy was quantified by counting the number of autophagosomes under each condition, averaged from 10 images per genotype for each condition (**Figures 4B–D** and Supplementary Figure 1B). Compared to the basal level of autophagy under control conditions, autophagy was significantly higher after stress treatments. In the presence of NAA, autophagy was still significantly induced by oxidative and ER stress conditions and in the presence of BTH, but no significant difference compared to control conditions was observed under nutrient starvation, salt and osmotic stresses. This suggests that NAA represses autophagy induced by sucrose and nitrogen starvation, salt and osmotic stresses, but not oxidative stress or ER stress, consistent with the results from overexpression of TOR.

To further confirm that addition of auxin represses stress-induced autophagy through activation of TOR, we examined whether auxin can inhibit the constitutive autophagy seen upon disruption of the TOR signaling pathway by chemical inhibition or genetic mutation (**Figures 4E–G**). To inhibit TOR kinase activity, 7-day-old *GFP-ATG8e* seedlings were transferred to liquid ½ MS medium with or without 20 nM NAA for 6–8 h, with DMSO or 1 μM AZD8055 added during the last 2–3 h of treatment. GFP-labeled autophagic bodies in roots after concanamycin A treatment were examined using confocal microscopy (**Figure 4A**). AZD8055 as expected led to a high accumulation of autophagic bodies in the vacuole, and NAA had no effect on this accumulation, suggesting that NAA acts upstream of TOR in the autophagy pathway. The extent of autophagy was quantified by counting root autophagosomes, and AZD8055 caused accumulation of autophagosomes both in the presence and absence of NAA, with no significant difference in autophagy induction (**Figure 4E**).

As an alternative approach, the effect of NAA upon inhibition of TOR complex function via knockout of *RAPTOR1B* was tested. Seven-day-old WT and *raptor1b* seedlings were transferred to liquid ½ MS medium with or without 20 nM NAA for 6–8 h, followed by MDC staining and autophagy detection by fluorescence microscopy (**Figures 4F,G**). NAA had no significant effect on the constitutive autophagy seen in the *raptor1b* mutant. Taken together, these results suggest that auxin acts upstream of TOR in the regulation of autophagy.

## Discussion

The TOR signaling pathway is a critical pathway for balancing cell growth and survival (Dobrenel et al., 2016). TOR was suggested to function as a complex with RAPTOR and LST8 based on studies in yeast and mammals (González and Hall, 2017), and previous studies in plants (Anderson et al., 2005; Deprost et al., 2005; Moreau et al., 2012). A knock out mutation in TOR is embryo-lethal in Arabidopsis (Menand et al., 2002), and down-regulation of TOR via RNA-interference arrests plant growth and induces autophagy. This suggests that TOR is a positive regulator of growth and development, and a negative regulator of autophagy in plants (Deprost et al., 2007; Liu and Bassham, 2010). To confirm that the TOR complex negatively regulates autophagy in Arabidopsis, we used a TOR inhibitor, AZD8055 (Chresta et al., 2010; Montane and Menand, 2013; Dong et al., 2015), which led to a significant induction of autophagy (**Figure 1A**). We also disrupted the TOR signaling pathway via a knockout mutant in *RAPTOR*, a binding partner of TOR. *RAPTOR1B* is the most highly expressed isoform of RAPTOR in Arabidopsis (Deprost et al., 2005), and *raptor1b* has a much more severe growth defect than *raptor1a* (Anderson et al., 2005). The *raptor1b* knockout line exhibited constitutive autophagy in both roots and leaf protoplasts, whereas a *raptor1a* mutation had only minor effects on autophagy, suggesting that RAPTOR1B is the primary RAPTOR isoform for repression of autophagy under our conditions.

Autophagy is induced by numerous stresses, including nutrient deficiency, salt, drought, oxidative and ER stresses (Doelling et al., 2002; Hanaoka et al., 2002; Xiong et al., 2007b; Liu et al., 2009, 2012). TOR has been well-characterized as regulating autophagy in response to nutrients in yeast and mammals, and down-regulation of TOR leads to growth defects and autophagy induction (Dobrenel et al., 2016). Therefore, we hypothesized that nutrient deficiency induces autophagy through the TOR signaling pathway in plants. As expected, overexpression of *TOR* repressed autophagy upon sucrose or nitrogen starvation, suggesting that TOR regulates nutrient deficiency-induced autophagy. Many upstream regulators of TOR have been identified in yeast and mammals, although many of them are not conserved in plants. One upstream kinase that is conserved throughout eukaryotes, named AMPK in mammals and Snf1 in yeast, senses energy status and activates autophagy in response to low energy (Hulsmans et al., 2016). A homolog of AMPK and Snf1 in plants, SnRK1, has been identified, and is also activated under stress conditions (Dobrenel et al., 2016; Nukarinen et al., 2016). SnRK1 phosphorylates RAPTOR1B, potentially decreasing TOR activity, although whether this affects autophagy is as yet unknown.

Salt and drought are two major environmental stresses encountered by plants; both lead to osmotic stress, while salt stress also leads to ionic stress. Surprisingly, overexpression of TOR and activation of TOR by auxin represses autophagy in both conditions, indicating that activation of autophagy upon salt and drought stress is also dependent on TOR. A substrate of TOR, S6K, shows reduced expression and activity under salt and osmotic stress (Mizoguchi et al., 1995; Mahfouz et al., 2006), suggesting that salt and osmotic stress reduce TOR activity. However, it is unclear how salt and osmotic stresses signal to the TOR signaling pathway. Salt, osmotic stress and nutrient deficiency all increase cellular ROS levels, which might function as signaling molecules or lead to oxidative stress (Zhu, 2016). A major source of signaling ROS is generated by plasma membrane NADPH oxidases (Miller et al., 2009), and we have shown previously that NADPH oxidase inhibitors block autophagy during nutrient deficiency and salt stress, but not osmotic stress (Liu et al., 2009). Osmotic stress activation of autophagy is therefore independent of NADPH oxidase. NADPH oxidase inhibitors also fail to inhibit the constitutive autophagy caused by down-regulation of *TOR* by RNA interference (Liu and Bassham, 2010). TOR may therefore act downstream of NADPH oxidase in regulating autophagy, or possibly in a parallel pathway that is independent of NADPH oxidase. SA has been shown to increase ROS signaling, and autophagy is induced by the SA analog BTH (Yoshimoto et al., 2009). However, overexpression of TOR or increasing TOR activity with auxin failed to inhibit BTH-induced autophagy, suggesting that SA-induced autophagy is TOR-independent. Excessive ROS also cause oxidative stress, and increasing TOR activity by overexpression or auxin addition failed to repress autophagy induced by H_2_O_2_, suggesting that oxidative stress activates autophagy through a TOR-independent pathway. It is still unclear whether signaling ROS regulate autophagy through TOR, and further work is needed to identify the stress sensors that trigger activation of autophagy.

Salt, drought, and heat stresses can also cause accumulation of excessive unfolded or misfolded proteins within the ER, known as ER stress. ER stress has been shown to induce autophagy in Arabidopsis (Liu et al., 2012; Yang et al., 2016). However, our data indicate that TOR overexpression has no effect during ER stress, suggesting that ER stress-induced autophagy is independent of TOR. Upon ER stress, the plant ER stress sensor inositol-requiring enzyme-1 (IRE1) splices the mRNA encoding the transcription factor membrane-associated basic leucine zipper 60 (bZIP60) to activate the unfolded protein response (UPR). The UPR aids proper folding or degradation of unfolded and misfolded proteins via upregulation of UPR-related genes (Howell, 2013). In Arabidopsis, induction of autophagy by ER stress is triggered by unfolded and misfolded proteins (Yang et al., 2016) and is dependent on one of the IRE1 isoforms, IRE1b, but not on IRE1a or bZIP60 (Liu et al., 2012). ER stress therefore appears to activate autophagy through IRE1b, in a pathway that is independent of TOR. However, how IRE1b regulates autophagy upon ER stress, and whether other UPR response genes are involved, requires further investigation.

Auxin has long been studied for its critical role in plant growth regulation (Enders and Strader, 2015). Auxin increases TOR activity, and auxin-mediated root gravitropism is impaired when TOR signaling is disrupted (Schepetilnikov et al., 2013). Auxin is unable to restore hypocotyl growth in estradiol-inducible *tor* mutants (Zhang et al., 2016), and many auxin response genes have reduced expression upon inhibition of TOR (Dong et al., 2015), suggesting that TOR is involved in auxin-regulated plant growth. Recent studies identified a small GTPase, ROP2, that mediates the activation of TOR by auxin (Li et al., 2017; Schepetilnikov et al., 2017). We hypothesized that enhancing TOR activity with auxin might repress stress-induced autophagy via the TOR signaling pathway. Indeed, as in the TOR overexpression lines, autophagy induced by nutrient starvation, salt or osmotic stresses was repressed by addition of NAA, whereas oxidative and ER stress-induced autophagy was not affected. NAA was unable to repress the autophagy induced by inhibition of TOR activity with the inhibitor AZD8055 or in a *raptor1b* knockout line. Exogenous application of the synthetic auxin 2, 4-D failed to restore growth of *raptor1b*, although *raptor1b* mutants can sense exogenous auxin normally (Anderson et al., 2005), supporting the conclusion that TOR signaling acts downstream of auxin.

In summary, we have demonstrated that autophagy can be regulated through TOR-dependent or -independent pathways, depending on the type of stress, and that auxin regulates plant stress responses through the TOR signaling pathway. Future work is required to identify the upstream stress sensors that repress TOR activity to allow activation of autophagy and the components of the TOR-independent autophagy activation pathway.

## Author Contributions

YP and DB designed the experiments. YP and XL conducted the experiments and analyzed data. YP and DB wrote the manuscript.

## Conflict of Interest Statement

The authors declare that the research was conducted in the absence of any commercial or financial relationships that could be construed as a potential conflict of interest.
