# Supplementary material for: TOR-Dependent and -Independent Pathways Regulate Autophagy in Arabidopsis thaliana
Source: Front Plant Sci. 2017 Jul 11;8:1204. doi: 10.3389/fpls.2017.01204 (PMC5504165; doi:10.3389/fpls.2017.01204)
Supplement: Supplementary file 1 [file Image_1.PDF]

Supplementary Material

TOR-dependent and –independent pathways regulate autophagy in *Arabidopsis thaliana*

Yunting Pu, Xinjuan Luo, Diane C. Bassham*

*** Correspondence:** Diane C. Bassham: bassham@iastate.edu

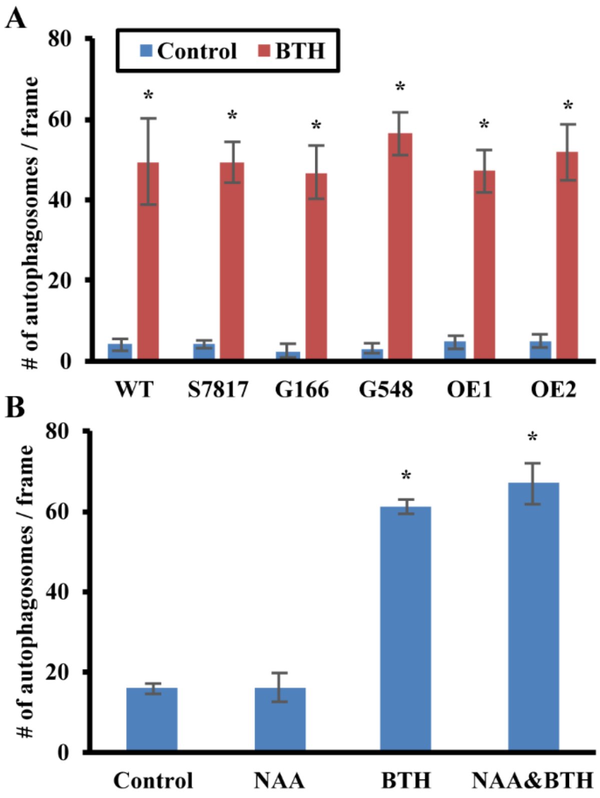

Supplementary Figure 1. Overexpression of TOR has No Effect on SA-Induced Autophagy

(A) 7-day-old seedlings of WT (Col-0) and *TOR* overexpression lines were treated with 80% ethanol (Control) or 100 µM BTH for 8 hours, stained with MDC and then observed and imaged by fluorescence microscopy. The number of puncta in each image was counted and averaged from 10 images per genotype for each condition. **(B)** 7-day-old *GFP-ATG8e* transgenic seedlings were treated with DMSO and 80% EtOH (Control), 20 nM NAA, 100 µM BTH or both NAA and BTH for 8 hours, then observed and imaged by fluorescence microscopy. The number of puncta in each image was counted and averaged from 10 images per condition. For both graphs, error bars indicate means ± SE from three independent replicates. Asterisks indicate statistically significant differences ($P < 0.05$) using Student's *t*-test compared with WT under control conditions.
